# Supplementary material for: Plastome Evolution and Comparative Analyses of a Recently Radiated Genus Vanda (Aeridinae, Orchidaceae)
Source: Int J Mol Sci. 2024 Sep 2;25(17):9538. doi: 10.3390/ijms25179538 (PMC11394697; doi:10.3390/ijms25179538)

Supplementary Materials

Table S1. The relative synonymous codon usage (RSCU) in plastomes of 13 *Vanda* species.

Table S2. Taxa, voucher and GenBank accession numbers of the species used in this study.

Figure S1. Comparison of junction between the LSC, SSC and IR regions among 13 *Vanda* plastomes.

Figure S2. Relative synonymous codon usage (RSCU) in the chloroplast genomes of 13 *Vanda* species and the amino acids encoded by these codons.

Figure S3. Global alignment of 13 *Vanda* plastomes using mVISTA with *Holcoglossum quasipinifolium* reference.

Figure S4. Maximum likelihood phylogenetic trees of *Vanda* species with four datasets.

Table S1. The relative synonymous codon usage (RSCU) in plastomes of 13 *Vanda* species.

**Codon**	***V. alpina***	***V. ampullaceum***	***V. brunnea***	***V. coelestis***	***V. coerulea***	***V. coerulescens***	***V. concolor***	***V. cristata***	***V. falcata***	***V. pumila***	***V. richardsiana***	***V. subconcolor***	***V. xichangensis***
UUU(F)	1.24	1.25	1.24	1.24	1.23	1.25	1.24	1.23	1.25	1.24	1.25	1.24	1.25
UUC(F)	0.76	0.75	0.76	0.76	0.77	0.75	0.76	0.77	0.75	0.76	0.75	0.76	0.75
UUA(L)	1.79	1.81	1.79	1.82	1.79	1.8	1.79	1.8	1.79	1.79	1.79	1.8	1.8
UUG(L)	1.34	1.33	1.34	1.34	1.35	1.34	1.34	1.35	1.35	1.34	1.35	1.34	1.34
CUU(L)	1.26	1.25	1.26	1.25	1.25	1.25	1.26	1.25	1.26	1.26	1.26	1.26	1.25
CUC(L)	0.4	0.41	0.4	0.38	0.4	0.39	0.41	0.41	0.39	0.4	0.39	0.4	0.4
CUA(L)	0.8	0.79	0.8	0.79	0.79	0.8	0.78	0.77	0.78	0.8	0.78	0.78	0.78
CUG(L)	0.42	0.43	0.42	0.42	0.43	0.42	0.42	0.43	0.43	0.42	0.43	0.42	0.43
AUU(I)	1.47	1.47	1.47	1.47	1.48	1.47	1.47	1.48	1.47	1.47	1.47	1.48	1.47
AUC(I)	0.65	0.65	0.65	0.65	0.65	0.65	0.65	0.64	0.64	0.65	0.64	0.64	0.64
AUA(I)	0.88	0.88	0.88	0.88	0.88	0.88	0.88	0.88	0.89	0.88	0.89	0.88	0.89
AUG(M)	1	1	1	1	1	1	1	1	1	1	1	1	1
GUU(V)	1.43	1.43	1.43	1.43	1.43	1.43	1.43	1.43	1.43	1.43	1.43	1.43	1.43
GUC(V)	0.54	0.54	0.54	0.54	0.54	0.54	0.54	0.54	0.54	0.54	0.54	0.54	0.54
GUA(V)	1.4	1.41	1.4	1.39	1.4	1.4	1.4	1.4	1.4	1.4	1.4	1.4	1.4
GUG(V)	0.63	0.63	0.63	0.63	0.63	0.63	0.63	0.63	0.63	0.63	0.63	0.63	0.63
UCU(S)	1.81	1.84	1.81	1.85	1.82	1.82	1.82	1.82	1.82	1.81	1.82	1.82	1.82
UCC(S)	1.03	1.02	1.03	1.01	1.04	1.02	1.02	1.03	1.03	1.03	1.03	1.02	1.03
UCA(S)	1.17	1.15	1.17	1.15	1.16	1.16	1.16	1.16	1.16	1.17	1.16	1.16	1.16
UCG(S)	0.48	0.48	0.48	0.48	0.48	0.49	0.49	0.48	0.48	0.48	0.48	0.49	0.47
CCU(P)	1.57	1.57	1.57	1.57	1.57	1.57	1.57	1.57	1.56	1.57	1.56	1.57	1.57

CCC(P)	0.91	0.91	0.91	0.91	0.91	0.91	0.91	0.9	0.91	0.91	0.91	0.91	0.9
CCA(P)	1.11	1.09	1.11	1.1	1.09	1.1	1.11	1.11	1.11	1.11	1.11	1.11	1.11
CCG(P)	0.41	0.43	0.41	0.43	0.43	0.42	0.42	0.42	0.42	0.41	0.42	0.42	0.42
ACU(T)	1.64	1.63	1.64	1.63	1.64	1.63	1.64	1.64	1.62	1.64	1.62	1.63	1.62
ACC(T)	0.68	0.68	0.68	0.68	0.68	0.69	0.69	0.68	0.7	0.68	0.7	0.69	0.7
ACA(T)	1.24	1.24	1.24	1.24	1.24	1.24	1.24	1.24	1.24	1.24	1.24	1.25	1.24
ACG(T)	0.43	0.45	0.43	0.44	0.44	0.43	0.43	0.44	0.44	0.43	0.44	0.43	0.44
GCU(A)	1.84	1.83	1.84	1.84	1.84	1.84	1.84	1.84	1.83	1.84	1.83	1.84	1.82
GCC(A)	0.56	0.56	0.56	0.56	0.56	0.56	0.56	0.56	0.57	0.56	0.57	0.56	0.56
GCA(A)	1.23	1.24	1.23	1.23	1.24	1.23	1.23	1.24	1.24	1.23	1.24	1.23	1.24
GCG(A)	0.37	0.37	0.37	0.37	0.36	0.37	0.37	0.36	0.37	0.37	0.37	0.37	0.37
UAU(Y)	1.61	1.62	1.61	1.6	1.6	1.61	1.6	1.61	1.61	1.61	1.61	1.6	1.61
UAC(Y)	0.39	0.38	0.39	0.4	0.4	0.39	0.4	0.39	0.39	0.39	0.39	0.4	0.39
UAA (*)	1.48	1.48	1.48	1.44	1.44	1.4	1.4	1.4	1.4	1.48	1.4	1.4	1.4
UAG (*)	0.82	0.82	0.82	0.86	0.9	0.9	0.9	0.9	0.9	0.82	0.9	0.9	0.9
CAU(H)	1.58	1.58	1.58	1.58	1.59	1.6	1.59	1.59	1.57	1.58	1.57	1.59	1.58
CAC(H)	0.42	0.42	0.42	0.42	0.41	0.4	0.41	0.41	0.43	0.42	0.43	0.41	0.42
CAA(Q)	1.5	1.5	1.5	1.5	1.5	1.5	1.49	1.5	1.5	1.5	1.5	1.5	1.5
CAG(Q)	0.5	0.5	0.5	0.5	0.5	0.5	0.51	0.5	0.5	0.5	0.5	0.5	0.5
AAU(N)	1.56	1.56	1.56	1.57	1.56	1.55	1.56	1.56	1.56	1.56	1.56	1.56	1.56
AAC(N)	0.44	0.44	0.44	0.43	0.44	0.45	0.44	0.44	0.44	0.44	0.44	0.44	0.44
AAA(K)	1.43	1.43	1.43	1.42	1.42	1.42	1.42	1.42	1.42	1.43	1.42	1.42	1.42
AAG(K)	0.57	0.57	0.57	0.58	0.58	0.58	0.58	0.58	0.58	0.57	0.58	0.58	0.58
GAU(D)	1.65	1.65	1.65	1.65	1.65	1.66	1.65	1.65	1.65	1.65	1.65	1.65	1.65
GAC(D)	0.35	0.35	0.35	0.35	0.35	0.34	0.35	0.35	0.35	0.35	0.35	0.35	0.35
GAA(E)	1.46	1.46	1.46	1.46	1.46	1.46	1.46	1.46	1.45	1.46	1.45	1.46	1.46

GAG(E)	0.54	0.54	0.54	0.54	0.54	0.54	0.54	0.54	0.55	0.54	0.55	0.54	0.54
UGU(C)	1.49	1.49	1.49	1.5	1.49	1.49	1.49	1.49	1.47	1.49	1.47	1.49	1.48
UGC(C)	0.51	0.51	0.51	0.5	0.51	0.51	0.51	0.51	0.53	0.51	0.53	0.51	0.52
UGA (*)	0.7	0.7	0.7	0.7	0.66	0.7	0.7	0.7	0.7	0.7	0.7	0.7	0.7
UGG(W)	1	1	1	1	1	1	1	1	1	1	1	1	1
CGU(R)	1.41	1.4	1.41	1.41	1.4	1.41	1.41	1.41	1.42	1.41	1.42	1.41	1.41
CGC(R)	0.32	0.32	0.32	0.32	0.33	0.32	0.32	0.32	0.31	0.32	0.31	0.32	0.32
CGA(R)	1.37	1.35	1.37	1.35	1.36	1.37	1.36	1.36	1.36	1.37	1.36	1.37	1.37
CGG(R)	0.39	0.4	0.39	0.39	0.4	0.39	0.4	0.4	0.4	0.39	0.4	0.4	0.4
AGU(S)	1.21	1.23	1.21	1.22	1.22	1.21	1.22	1.22	1.22	1.21	1.22	1.21	1.22
AGC(S)	0.29	0.29	0.29	0.3	0.29	0.29	0.29	0.3	0.29	0.29	0.29	0.29	0.29
AGA(R)	1.86	1.89	1.86	1.89	1.87	1.88	1.88	1.86	1.87	1.86	1.87	1.88	1.87
AGG(R)	0.64	0.65	0.64	0.63	0.64	0.63	0.63	0.64	0.63	0.64	0.63	0.63	0.63
GGU(G)	1.33	1.32	1.33	1.32	1.32	1.32	1.33	1.32	1.32	1.33	1.32	1.33	1.32
GGC(G)	0.39	0.4	0.39	0.39	0.4	0.39	0.39	0.4	0.39	0.39	0.39	0.39	0.4
GGA(G)	1.58	1.57	1.58	1.58	1.58	1.59	1.57	1.58	1.58	1.58	1.58	1.58	1.59
GGG(G)	0.7	0.71	0.7	0.7	0.7	0.7	0.7	0.7	0.7	0.7	0.7	0.7	0.7

Table S2. Taxa, voucher and GenBank accession numbers of the species used in this study.

	Species	Voucher	GenBank accession No.
Ingroups	*Vanda alpina*	LY & XXG, 2021853, Yunnan, China (cultivated in XTBG)	PQ009216
	V. ampullaceum	LY & XXG, 2021850, Yunnan, China (cultivated in XTBG)	PQ009217
	V. coerulea	LY, Yunnan, China (cultivated in XTBG)	PQ009218
	V. cristata	LY & XXG, 2021852, Yunnan, China (cultivated in XTBG)	PQ009219
	V. pumila	LY & XXG, 2021842, Yunnan, China (cultivated in XTBG)	PQ009220
	V. brunnea	-	NC_041522
	V. coelestis	-	NC_086850
	V. coerulescens	-	MN711650
	V. concolor	-	NC_048458
	V. falcata	-	NC_036372
	V. richardsiana	-	NC_036373
	V. subconcolor	-	NC_047413
	V. xichangensis	-	NC_047197
Outgroups	*Holcoglossum quasipinifolium*	-	NC_041516
	Luisia morsei	-	NC_086699

Figure S1. Comparison of junction between the LSC region, SSC region and IR regions among 13 *Vanda* plastomes. The topology and taxon orders were consistent to Figure 5.

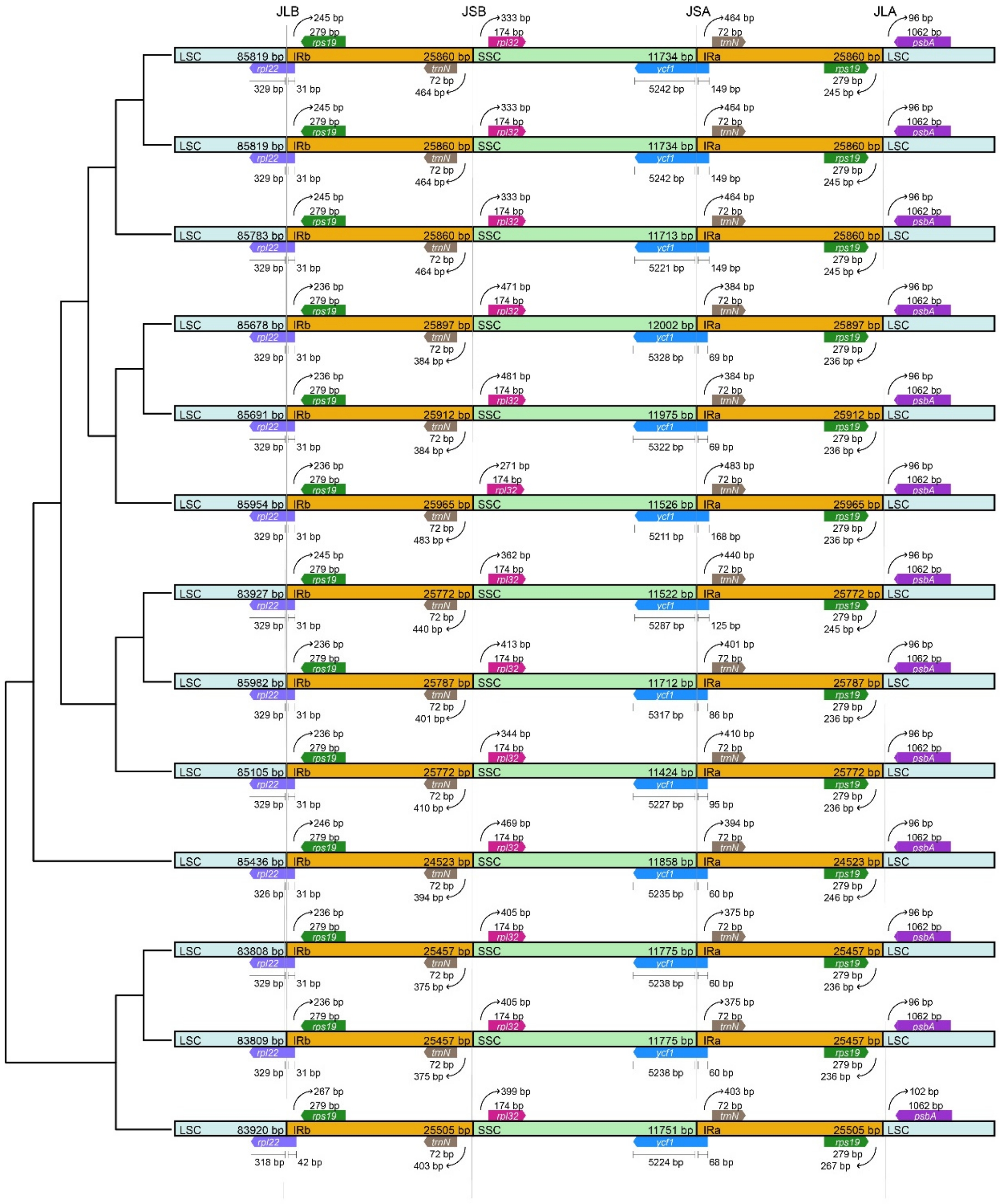

Figure S2. Relative synonymous codon usage (RSCU) in the plastomes of 13 *Vanda* species and the amino acids encoded by these codons. Color key: the red values mean higher RSCU values and the blue values mean lower RSCU values.

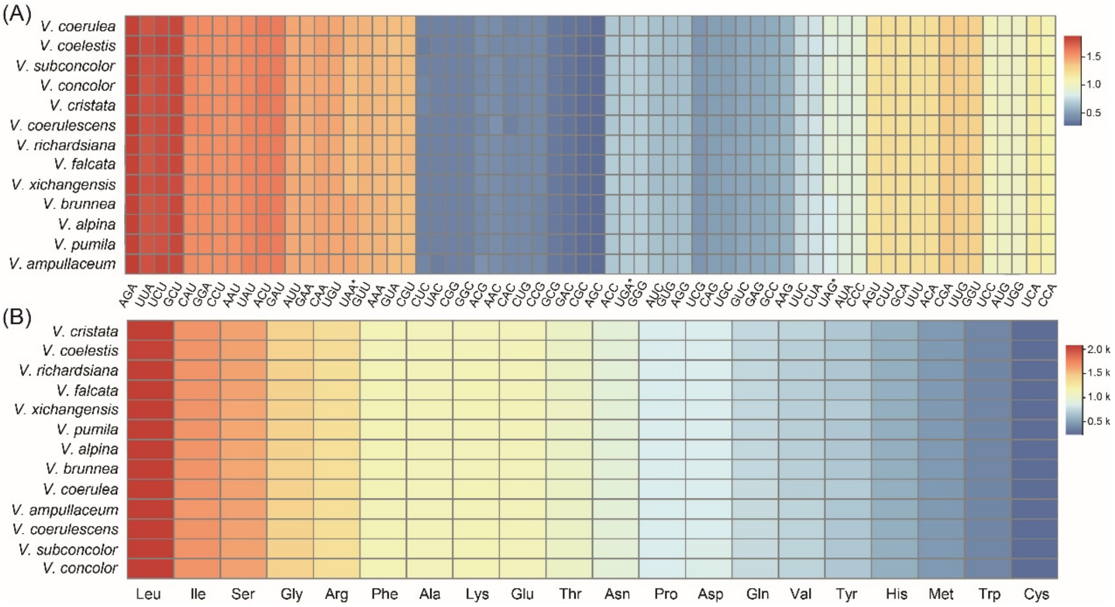

Figure S3. Global alignment of 13 *Vanda* plastomes using mVISTA with *Holcoglossum quasipinifolium* reference. The y-axis shows the coordinates between the plastomes. The topology and taxon orders were consistent to Figure 5.

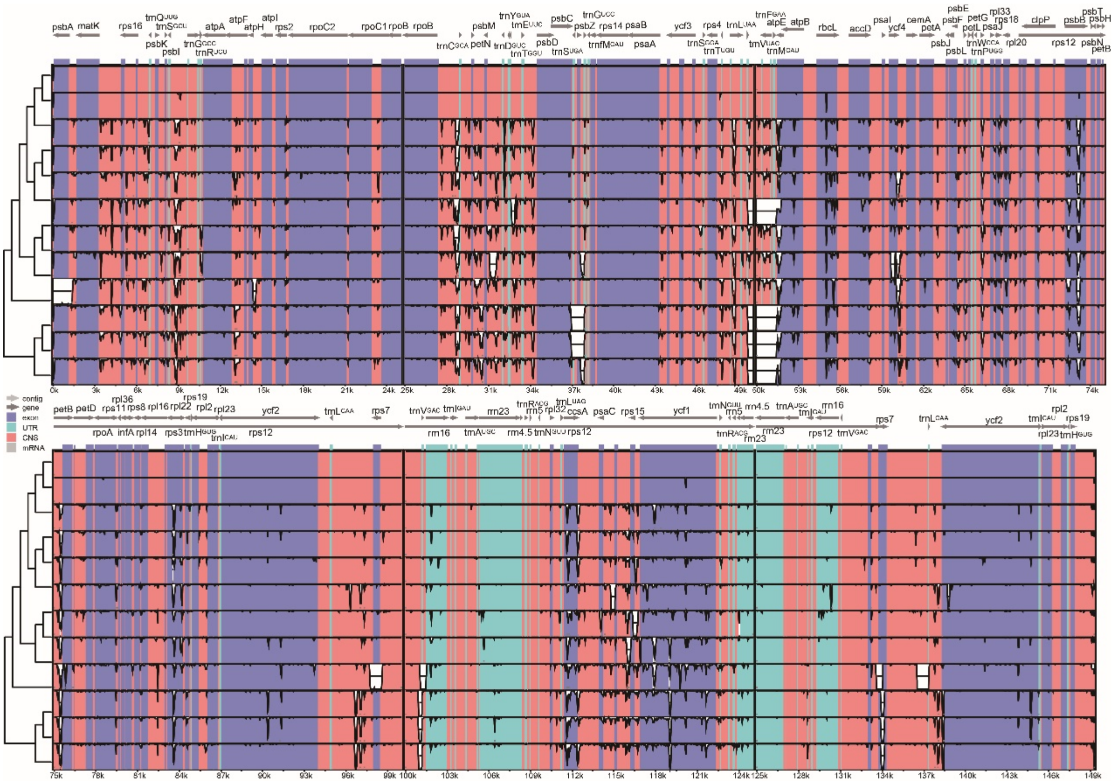

Figure S4. Maximum likelihood phylogenetic trees of *Vanda species* with four datasets. (A) phylogenetic topology from LSC, (B) phylogenetic topology from 68 CDSs; (C) phylogenetic topology from five hypervariable non-CDSs; and (D) phylogenetic topology from six hypervariable CDSs. The numbers above the branches represented the supporting values from Bayesian inference and maximum likelihood analyses, respectively.

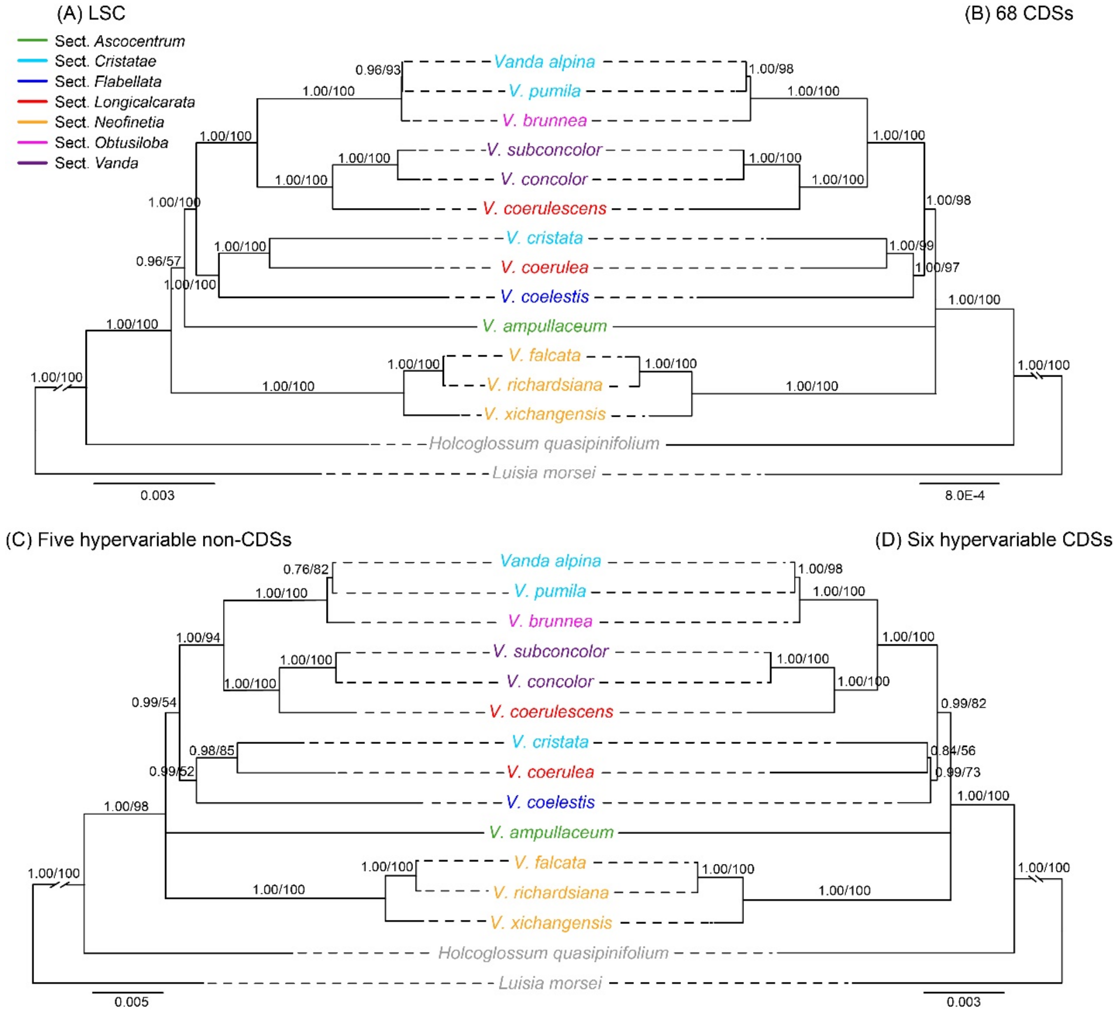

Supplement: Supplementary file 1 [file ijms-25-09538-s001.zip › ijms-3149424-supplementary.pdf]
